# Water-Tree Characteristics and Its Mechanical Mechanism of Crosslinked Polyethylene Grafted with Polar-Group Molecules

**DOI:** 10.3390/ijms23169450

**Published:** 2022-08-21

**Authors:** Xiao-Xia Zheng, You-Cheng Pan, Wei-Feng Sun

**Affiliations:** 1 College of Computer Science and Technology, Heilongjiang Institute of Technology, Harbin 150050, China; 2 Key Laboratory of Cold Region Urban and Rural Human Settlement Environment Science and Technology, Ministry of Industry and Information Technology, School of Architecture, Harbin Institute of Technology, Harbin 150006, China; 3 School of Electrical and Electronic Engineering, Nanyang Technological University, Singapore 639798, Singapore

**Keywords:** crosslinked polyethylene, polar-group molecule, peroxide graft, water-tree resistance

## Abstract

In order to restrain electric-stress impacts of water micro-droplets in insulation defects under alternating current (AC) electric fields in crosslinked polyethylene (XLPE) material, the present study represents chemical graft modifications of introducing chloroacetic acid allyl ester (CAAE) and maleic anhydride (MAH) individually as two specific polar-group molecules into XLPE material with peroxide melting approach. The accelerated water-tree aging experiments are implemented by means of a water-blade electrode to measure the improved water resistance and the affording mechanism of the graft-modified XLPE material in reference to benchmark XLPE. Melting–crystallization process, dynamic viscoelasticity and stress-strain characteristics are tested utilizing differential scanning calorimeter (DSC), dynamic thermomechanical analyzer (DMA) and electronic tension machine, respectively. Water-tree morphology is observed for various aging times to evaluate dimension characteristics in water-tree developing processes. Monte Carlo molecular simulations are performed to calculate free-energy, thermodynamic phase diagram, interaction parameter and mixing energy of binary mixing systems consisting of CAAE or MAH and water molecules to evaluate their thermodynamic miscibility. Water-tree experiments indicate that water-tree resistance to XLPE can be significantly improved by grafting CAAE or MAH, as indicated by reducing the characteristic length of water-trees from 120 to 80 μm. Heterogeneous nucleation centers of polyethylene crystallization are rendered by the grafted polar-group molecules to ameliorate crystalline microstructures, as manifested by crystallinity increment from 33.5 to 36.2, which favors improving water-tree resistance and mechanical performances. The highly hydrophilic nature of CAAE can evidently inhibit water molecules from aggregating into water micro-droplets in amorphous regions between crystal lamellae, thus acquiring a significant promotion in water-tree resistance of CAAE-modified XLPE. In contrast, the grafted MAH molecules can enhance van der Waals forces between polyethylene molecular chains in amorphous regions much greater than the grafted CAAE and simultaneously act as more efficient crystallization nucleation centers to ameliorate crystalline microstructures of XLPE, resulting in a greater improvement (relaxation peak magnitude increases by >10%) of mechanical toughness in amorphous phase, which primarily accounts for water-tree resistance promotion.

## 1. Introduction

Water-tree aging in polymeric insulation materials has attracted considerable attention due to the inevitable moisture and water-bath environments that cause insulation failures under long-term mechanical fatigues. Nevertheless, there is, so far, no comprehensive explanation and clarified mechanism of water-tree initiation and propagation. Insulation defects and internal electric field distortion in crosslinked polyethylene (XLPE) material under thermal-water environments are dominant triggers leading to water-tree aging of electric insulation [1,2]. The electric field strength at water-tree terminals gradually increases with aging time, resulting in serious local discharge, which forms electric-trees and finally causes dielectric breakdown of XLPE main insulation in power cables. Alternating current (AC) electric field is more likely to render water-trees than DC electric field. The frequency of AC electric fields is one important factor accounting for water-trees, and the electrical, chemical and mechanical characteristics are comprehensively related to water-tree resistant performance of polymeric insulation materials. At present, mechanical damage and electrochemical oxidation are the two most recognized fundamental mechanisms for water-tree initiation and propagation [3].

Electromechanical stress theory is the focus of mechanical damage theory, indicating that the macroscopic molecular-chains of XLPE material are not neatly arranged and thus give rise to substantial holes and frail areas where periodic elastic deformations (local motions of polymer molecular-chains) frequently occur, causing mechanical fatigue under Maxwell’s stress derived from AC electric field [4]. Moreover, XLPE molecular-chains will be oriented under the electric field greater than 30 MV/m at water-tree terminal [5]. Meanwhile, the orientation behavior and thermal motions of polymer molecular-chains are two contrary processes in opposite trends, in which the thermal motions of polymers intensify with increasing temperature in disfavor of orientation behaviors. In semi-crystalline polymers, such as XLPE, there are large numbers of molecular branches in amorphous regions, which are in irregular arrangements and will be oriented under AC electric field. The molecular-chains perpendicular to the orientation direction subjected to Maxwell stress is more prone to break under electric-stress fatigue than the unoriented molecular-chains, thus forming micro-cracks along orientation direction in amorphous regions, which accounts for the higher water-tree propagation speed in the electric field direction than the other directions.

Due to the remarkable discrepancy in dielectric permittivity, the water molecules will undergo electrostrictive actions under electric field, rendering local pressures at insulation defects in polyethylene amorphous regions [6]. Accordingly, the amorphous molecular-chains of XLPE suffer continuous impacts of water micro-droplets filled in micro-cracks under AC electric-stress to be further broken, leading to the enlargement of micro-cracks full of water micro-droplets and forming water-fill channels, which will randomly extend along electric field [7]. Compared to amorphous regions, the polyethylene molecular-chains assemble into condensed periodic lattices with much higher stability in crystalline regions, so water-trees are only initiated and propagate in amorphous regions of XLPE material [8]. Water-trees consist of tree-shaped channels of water-filling defects produced inside XLPE insulation under electric field in water-bath environments, which is classified into bow-tie and tube types according to their macroscopic shape [9]. The condition of producing water-trees resides in the micro-droplets forming at structural defects, which should be inhibited for alleviating water-tree aging [10,11]. Three-dimensional crosslinking networks in XLPE amorphous regions can restrict the impacts of micro-droplets on amorphous molecular-chains from forming water-trees when local deformation occurs along electric field direction, which essentially accounts for the high water-tree resistance of XLPE material [12,13]. It has been reported that the larger size and fewer grains of XLPE spherulites conduce to a higher speed of water-tree propagation [14]. Employing elastomer (SEBS and EVA), inorganic nanofiller (nano MgO) or auxiliary crosslinker (HAV2) for XLPE modifications can ameliorate crystallization morphology, increase crystallinity or crosslink-degree so as to improve water-tree resistance and electrical insulation performances of XLPE material [8,15,16,17].

By grafting polar-group molecules, the hydrophilicity of XLPE molecular-chains can be significantly improved to restrain water molecules in structural defects from aggregating into micro-droplets but resolve them into water membranes, which will inhibit growth of water-trees. Both chloroacetic acid allyl ester (CAAE) and maleic anhydride (MAH) molecules containing multiple polar groups as well as vinyl group, which should be competent for grafting on polyethylene molecular-chains to fulfill molecular-level modifications on XLPE material, are expected to acquire a substantial improvement in water-tree resistance. In the present study, by the melting blend method and thermal chemistry, we make chemical graft modifications on XLPE material with CAAE and MAH, focusing on improving water-tree resistant performance and trying to reveal the correlated modification mechanism, which is expected to provide strategic technical support for developing water-tree resistant XLPE materials for power cables.

## 2. Results and Discussion

### 2.1. Infrared Spectroscopic Analysis

**Figure 1:**
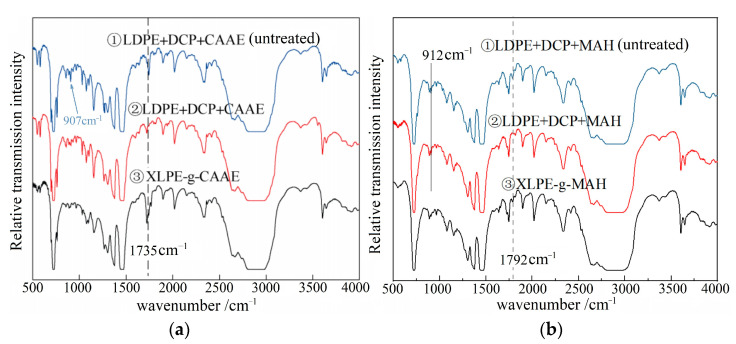
Fourier-transform infrared spectra of uncrosslinked raw material blends before and after vacuum hot-degassing (① and ②) and the modified XLPE (③): (**a**) grafting 1.0 wt% CAAE and (**b**) grafting 1.0 wt% MAH.

The successful modification of chemical grafting on XLPE can be verified by FTIR spectroscopy, which is characterized by stretching vibration peaks at 907 and 1735 cm^−1^ of vinyl (C=C) and ester (C=O) groups on CAAE and at 912 cm^−1^ of MAH molecules [18,19], as shown in Figure 1. In comparison to both the raw material blends before and after vacuum hot-degassing treatment, the CAAE-grafted XLPE (XLPE-g-CAAE) with 1.0 wt% grafting content gives rise to the characteristic peak of C=O groups. The infrared absorption peak at 907 cm^−1^ of C=C groups in CAAE molecules arises in the mixtures of LDPE, DCP and CAAE without crosslinking and grafting reactions. By contrast, the characteristic peak of C=C disappears in XLPE-g-CAAE, demonstrating that CAAE molecules have been successfully grafted onto XLPE molecular-chains. Moreover, the characteristic absorption peak at 912 cm^−1^ of unsaturated chemical bonds in MAH molecules disappears in MAH-grafted XLPE (XLPE-g-MAH) with 1.0 wt% grafting content, giving rise to a new peak at 1792 cm^−1^ of carbonyl groups after grafting reaction, indicating the successful graft of MAH molecules to XLPE. These FTIR results are also identically presented for the other modified XLPE materials with grafting contents of 0.5 and 1.5 wt%. Therefore, it has been verified by FTIR spectroscopy that chemical modifications of grafting CAAE and MAH to XLPE have been fulfilled.

### 2.2. Water-Tree Morphology and Growth Length

**Figure 2:**
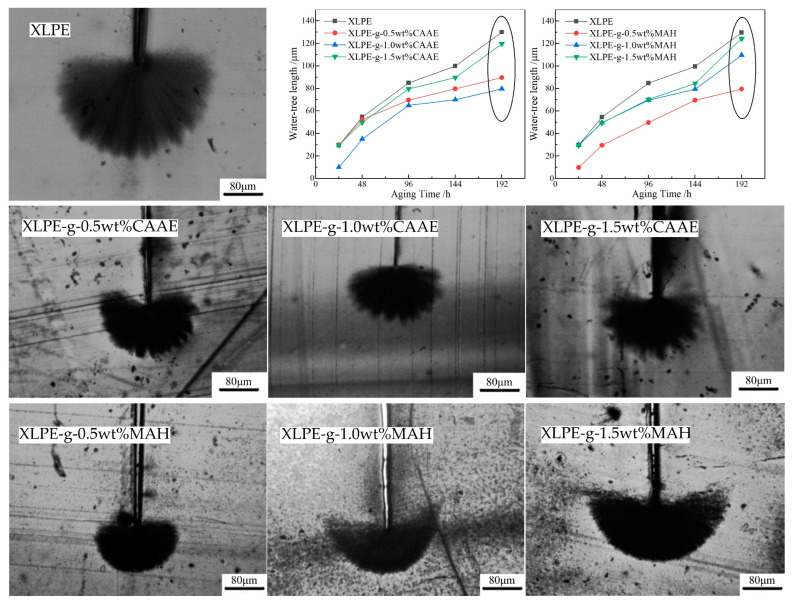
Water-tree morphology observed with optical microscopy for XLPE modified by grafting CAAE or MAH (image photos), and water-tree length versus aging time for XLPE-g-CAAE and XLPE-g-MAH compared with benchmark XLPE (top-right curve profiles).

Taking the ungrafted XLPE material as a benchmark, the water-tree morphologies and growth lengths of XLPE-g-CAAE and XLPE-g-MAH with grafted contents of 0.5, 1.0 and 1.5 wt% (XLPE-g-wt%CAAE and XLPE-g-wt%MAH) are observed and evaluated by optical microscopy, as illustrated by photo images of water-trees aging for 192 h and by water-tree length versus aging time profiles in Figure 2. Water-tree resistant performance is characterized by water-tree length at the same aging time, which means the smaller water-tree length represents the higher water-tree resistance. As shown by curve profiles in the top-right two panels of Figure 2, the water-tree lengths of all the tested samples increase with aging time. When the CAAE grafting content reaches 1.0 wt%, the water-tree resistance is improved most significantly, as manifested by water-tree lengths for aging longer than 48 h. In addition, XLPE-g-1.5wt%CAAE are more severe for water-tree aging than XLPE-g-0.5wt%CAAE.

Polar groups on the grafted CAAE and MAH molecules evidently promote the hydrophilicity of XLPE so that the water molecules penetrating into XLPE amorphous regions are mostly adsorbed near these polar groups and disfavor aggregating into micro-droplets at structural defects, which alleviates the impacts of electrostrictive water micro-droplets on polyethylene molecular-chains, as manifested by the appreciable inhibition of water-tree propagation. Meanwhile, the grafted molecules increase molecular-chain density in XLPE amorphous regions, which also accounts for improving water-tree resistance. Further, the excessively high grafting content of CAAE leads to incompatible interfaces in XLPE, which will produce structural defects that initiate water-tree and benefit water-tree propagation, thereby reducing water-tree resistance instead. In contrast, when the grafting content of MAH exceeds 0.5 wt%, the inhibition of water-tree aging fades away, indicating the different underlying mechanisms of grafting CAAE and MAH for restraining water-tree aging that will be further revealed in the following sections.

### 2.3. Melting–Crystallization Characteristics

**Figure 3:**
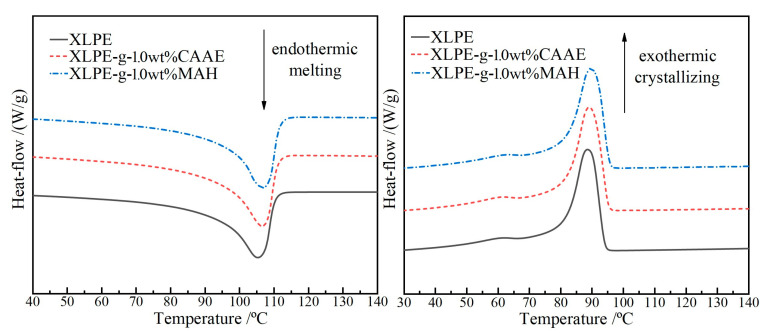
DSC temperature spectra of XLPE, XLPE-g-1.0wt%CAAE and XLPE-g-1.0wt%MAH in melting (**left** **panel**) and crystallizing (**right panel**) processes.

Melting–crystallization characteristics of XLPE benchmark, XLPE-g-1.0wt%CAAE and XLPE-g-1.0wt%MAH (as the paradigm of graft-modified XLPE) are represented by endothermic and exothermic heat-flows of DSC temperature spectra in reverse processes of heated-melting and cooled-crystallization to evaluate semi-crystalline crystallinity by comparing melting and crystallizing peaks, as shown in Figure 3 and Table 1. In contrast to benchmark XLPE, the modified XLPE grafted with CAAE or MAH acquire substantial elevations in both crystallizing and melting temperatures and in crystallinity, implying that the polar groups on the grafted molecules lead to higher intermolecular forces between polyethylene molecular-chains. Meanwhile, the grafted molecules undergo as the heterogeneous nucleation centers for polyethylene molecular-chain crystallization, which accounts for the smaller size and higher density of spherulites in XLPE microscopic crystallization structure, resulting in the reductions of the spacing between lamellae and the volume of amorphous regions.

The initial increase slope in crystallizing peak indicates crystallization nucleation rate, whilst the difference between initial temperature and peak temperature characterizes the inverse rate of crystal growth, which are, respectively, higher and lower for the graft-modified XLPE than that for XLPE benchmark. It is thus verified from DSC tests that both the nucleation rate and crystal growth rate of the graft-modified XLPE are higher than those of XLPE benchmark. It is hereby verified that the polar-group molecules (especially for MAH) grafted on polyethylene molecule-chains are capable of enhancing van der Waals interactions between polyethylene molecule-chains and acting as heterogeneous nucleation centers to expedite crystallization nucleation and crystal growth, resulting in higher densities of lamellae and spherulites, as manifested by the higher crystallization enthalpy and crystallinity, respectively.

**Figure 4:**
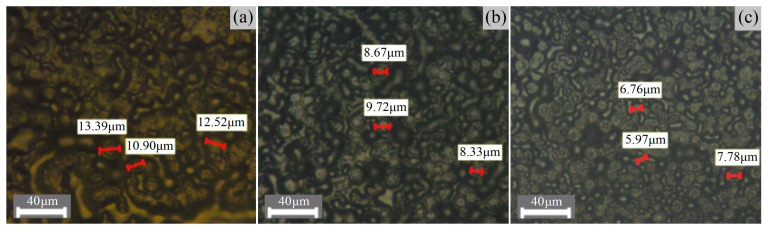
Spherulitic semi-crystalline morphologies of (**a**) XLPE, (**b**) XLPE-g-1.0wt%CAAE and (**c**) XLPE-g-1.0wt%MAH observed by PLM.

Optical microscopic images of semi-crystalline morphologies with spherullites and amorphous regions in XLPE, XLPE-g-1.0wt%CAAE and XLPE-g-1.0wt%MAH are shown in Figure 4. Compared with XLPE benchmark, the grafted XLPE materials represent a higher density of spherulites in smaller diameters, resulting in a considerably smaller volume ratio of amorphous phase, for which XLPE-g-MAH is more evident than XLPE-g-CAAE. Based on crystallization kinetics, the polyethylene lamellae are formed by folding regular molecular-chains, while a spherulite is formed by a large number of lamellae arranged radically from a crystallizing nucleus. Therefore, the grafted CAAE or MAH molecules could act as crystallization nuclei to initiate the crystallization of polyethylene lamellae, which will finally develop into spherulites, resulting in a higher crystallinity as manifested by a higher density of smaller-sized spherulites.

### 2.4. Viscoelasticity and Stress-Strain Characteristics

**Figure 5:**
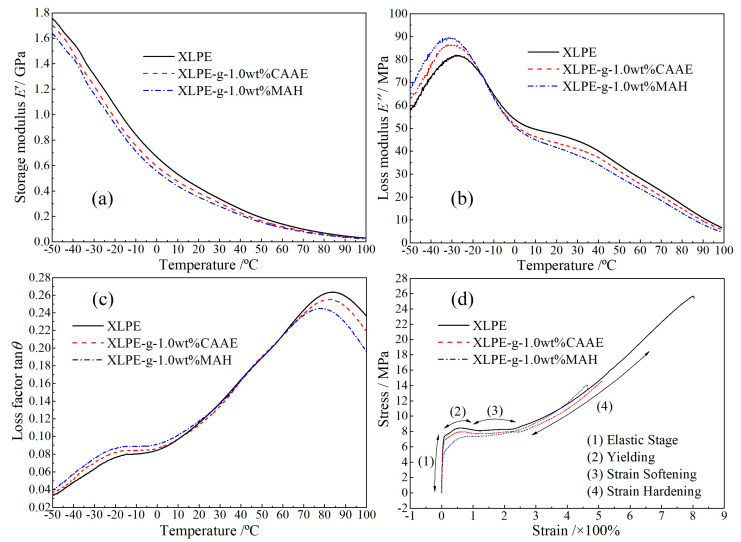
DMA temperature spectra of (**a**) storage modulus, (**b**) loss modulus, (**c**) loss factor and (**d**) stress-strain characteristics for XLPE-g-1.0wt%CAAE and XLPE-g-1.0wt%MAH in comparison to XLPE benchmark.

In dynamic relaxation temperature spectra of DMA, the storage modulus *E*’ indicates the mechanical stiffness of polymer materials, while loss modulus *E*” and loss factor tan*θ* characterize the mechanical toughness of polymer materials. DMA temperature spectra and static tensile characteristics of 1.0wt%-graft XLPE in reference to XLPE are shown in Figure 5a–c, with the peak magnitudes and water-tree lengths (aging for 192 h) listed in Table 2. Mechanical toughness of amorphous regions can be measured from the intensities of *E*” and tan*θ* peaks at glass-transition temperature (*β* relaxation peak at −25 °C) [20,21], which are dominantly derived from the relaxations of amorphous molecular-chains connecting to lamella surfaces. As the density of lamellae in XLPE increases, the *β* relaxation peak shifts toward a lower temperature, and the peak intensity of *E*” increases, implying that the molecular-chain relaxations on lamella surfaces are intensified. The *β* peak intensities of *E*” and tan*θ* for XLPE-g-1.0wt%CAAE and XLPE-g-1.0wt%MAH are distinctly higher than that for XLPE benchmark, in which XLPE-g-1.0wt%MAH shows the highest values. Therefore, the mechanical toughness in amorphous regions between the lamellae of XLPE material is enhanced by grafting CAAE and MAH molecules, which improves the resistance to the electric-stress damage from micro-droplets on polyethylene molecular-chains, as manifested consistently by the higher crystallinities.

The tan*θ* peaks appearing at the higher temperature of 80 °C (*α* relaxation peak) come from the rotation and slip of lamellae and the relaxation of fold molecular-chains on lamella surfaces [22]. The graft of polar-group molecules ameliorates crystalline characteristics by reducing the volume ratio of XLPE amorphous phase and the size of spherulites, resulting in the effective restriction on *α* relaxation, which accounts for the lowest *α* peak of XLPE-g-1.0wt%MAH. Although XLPE-g-1.0wt%MAH shows the highest crystallinity and mechanical toughness, its water-tree resistant performance is similar to XLPE-g-1.0wt%CAAE (as shown in Figure 2), implying a discrepant mechanism for improving water-tree resistance by grafting CAAE.

Stress-strain characteristics include four stages during dynamic mechanical tensile process: elastic, yielding, strain-softening and strain hardening stages, as shown in Figure 5d. Because the grafting reaction causes breakages in parts of polyethylene molecular-chains, the fracture stress and elongation of the graft-modified XLPE materials are distinctly lower than that of benchmark XLPE [23,24]. After grafting modifications, both elastic modulus and yield strength decrease, whilst strain softening and elastic (cold stretching) processes are shortened, and even XLPE-g-1.0wt%MAH exhibits no perceptible strain softening stage. Amorphous regions between lamellae in XLPE material are diminished by chemically introducing polar-group molecules, which restricts the slips between lamellae, as indicated by the shortened strain softening and cold stretching processes.

### 2.5. Miscibility of Grafted Molecules with Water

**Figure 6:**
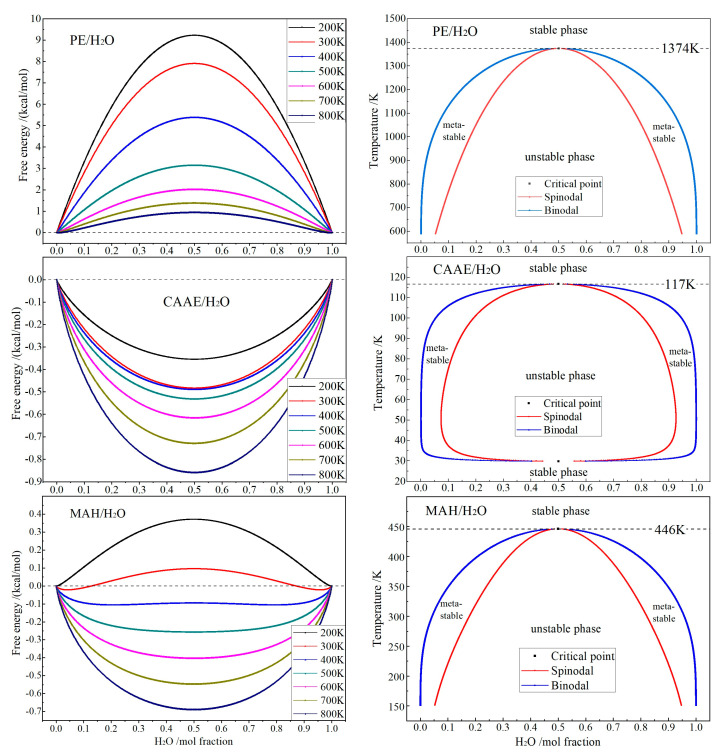
Free energy (**left** **panels**) at 200–800 K and phase diagram (**right** **panels**) of PE/H_2_O, CAAE/H_2_O and MAH/H_2_O binary molecular mixtures.

Employing the modified Flory–Huggins model [25] for the thermodynamics of binary molecular-mixture systems, the free energy, phase diagram and mixing energy of PE/H_2_O, CAAE/H_2_O and MAH/H_2_O binary molecular mixtures are calculated to analyze the hydrophilicity of polyethylene molecular-chains and polar-group molecules, as shown in Figure 6. Although the solubility of H_2_O in polyethylene (PE) is very low and the critical point temperature reaches 1374K, the metastable phase region located between Spinodal and Binodal boundaries remains largely in phase diagram, implying that ~5 mol% of H_2_O molecules will penetrate into polyethylene amorphous regions to form a metastable mixture above 600 K.

Due to the existence of polar groups, both CAAE and MAH molecules represent much higher hydrophilic features than PE molecules, with the critical point temperatures approaching 117 and 446 K, respectively. In contrast, the molecular compatibility of CAAE with H_2_O is significantly higher than that of MAH, and the critical point temperature is lower than room temperature, implying that CAAE and water can completely dissolve with each other in any proportion. Therefore, it is suggested that water is able to exist as molecules around MAH and CAAE in graft-modified materials, but water molecules cannot disperse uniformly and will aggregate into water droplets to form phase separation around polyethylene molecular chains without polar groups.

**Figure 7:**
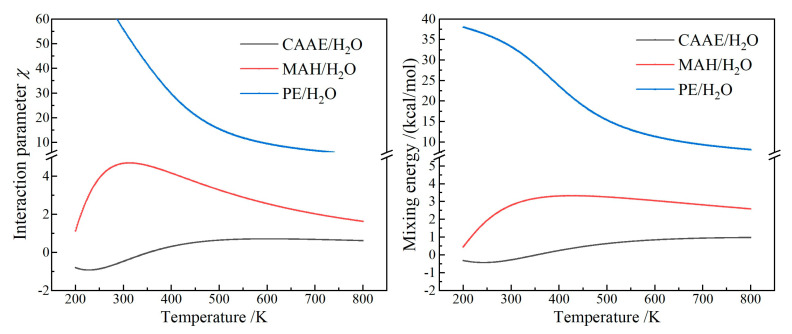
Interaction parameter (**left panel**) and mixing energy (**right panel**) of PE/H_2_O, CAAE/H_2_O and MAH/H_2_O binary molecular mixtures.

A small or negative interaction parameter (*χ*) indicates that the two molecular components interact strongly at a specific temperature to form a binary mixing system under thermodynamic equilibrium. While for a large positive *χ*, the two different molecules prefer to cluster with their peers to form a separate two-phase system. Interaction parameters and mixing energies (*E*_mix_) of PE/H_2_O, CAAE/H_2_O and MAH/H_2_O binary mixing systems as a function of temperature are shown in Figure 7. Binary mixing systems of CAAE/H_2_O and MAH/H_2_O present a higher magnitude of *χ* and *E*_mix_ than PE/H_2_O. The *χ* and *E*_mix_ of CAAE/H_2_O are negative at temperatures below 350 K, while MAH/H_2_O represents two positive parameters in temperature range of 200–800 K, approaching maximum at 300 K, which is remarkably higher than that of CAAE/H_2_O. Since the polar-group molecules in graft-modified XLPE materials are all chemically bonded to polyethylene molecular-chains, the water molecules infiltrated into XLPE-g-CAAE prefer to disperse and dissolve around polar groups rather than assembling into micro-droplets.

### 2.6. Mechanism of Water-Tree Resistance

According to the melting–crystallization characteristics and DMA temperature spectra with the derived water-tree resistant mechanisms introduced by grafting polar-group molecules, it is reasonable to predict that besides improving water resistance by ameliorating crystallization structure of XLPE, the imported hydrophilia of polar groups can restrain water molecules from aggregating into micro-droplets, resulting in improved water-tree resistance. Macroscopic molecular-chains of XLPE pass through several crystallization regions and tangle with each other. Hence, polyethylene molecular-chains in amorphous phase, which determine slides between lamellae, undergo deformations and relaxation motions under electric-stress impacts of water micro-droplets. When semi-crystalline XLPE materials bear a mechanical stretching process in strain hardening stage, the molecular-chains connecting lamellae in amorphous regions mainly bear the applied mechanical forces, which means the stronger connecting molecular-chains or the smaller amorphous regions require a higher external force to produce macroscopic deformations.

**Figure 8:**
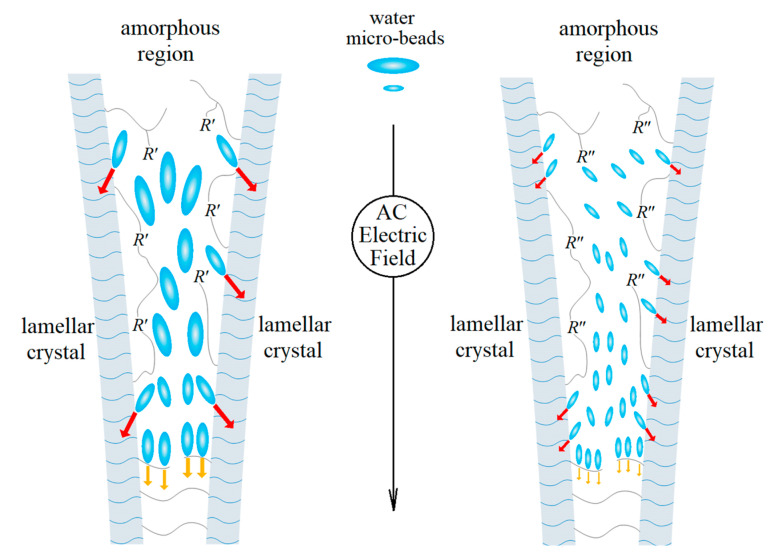
Schematic electromechanical mechanism of water micro-droplets breaking through the amorphous regions in XLPE-g-CAAE (**left** **panel**) and XLPE-g-MAH (**right** **panel**).

Although XLPE-g-MAH has a greater strain hardening strength and higher/lower *β*/*α* relaxation peaks (as shown in Figure 5), the hydrophilicity of CAAE far exceeds MAH, so XLPE-g-CAAE possesses a nearly identical water-tree resistance as XLPE-g-MAH. The grafted hydrophilic CAAE can effectively disperse water micro-droplets gathered in amorphous regions between lamellae, thus alleviating electrical-mechanical damage of water micro-droplets on defect areas under AC electric field, as shown in Figure 8, in which *R*’ and *R*” respectively denote the grafted MAH and CAAE on polyethylene molecular-chains. In contrast to XLPE-g-MAH, the water micro-droplets in amorphous regions of XLPE-g-CAAE are much smaller, which will not further aggregate in insulation defects to form water-filling holes and thus are difficult to render water-tree channels.

## 3. Methods and Materials

### 3.1. Material Preparation

Raw materials for the melting blend process are comprised of dicumyl peroxide (DCP, Nobel Company Ltd., Aksu, China) as the crosslinking and grafting initiator, low-density polyethylene (LDPE, LD200GH, Sinopec Company Ltd., Beijing, China) as the basis material, and chloroacetic acid allyl ester or maleic anhydride (CAAE or MAH, Ruierfeng Chemical Co. Ltd. Guangzhou, China) as the grafting modification agents. In melting blend process of preparing initial mixture materials before crosslinking and grafting reactions, the pristine LDPE material is heated for melting at 110 °C temperature for 3 min with a rotating speed of 40 rpm in torque rheometer (RM200C, Hapro Co. Ltd., Harbin, China), and then 2 wt% DCP and 0.3 wt% 1010 antioxidant together with 0, 0.5 and 1.0 wt% CAAE or MAH are added into torque rheometer blending for 17 min and cooled down to room temperature, thus obtaining the uniformly mixed material. For crosslinking/grafting chemical reactions, the prepared blend is first heated to 120 °C for melting in plate vulcanizer, and then is further heated to 175 °C at a rate of 5 °C/min with the pressure being raised to 15 MPa by a rate of 1 MPa/min. After 35 min, the crosslinked and grafted polyethylene material is cooled down to room temperature and then compressed into molding films. Eventually, the film samples are hot-degassed under short-circuit in vacuum drying chamber at 80 °C for 48 h so as to clear off the residual reactants and reaction by-products and to relax mechanical stresses in samples.

### 3.2. Water-Tree Experiment

**Figure 9:**
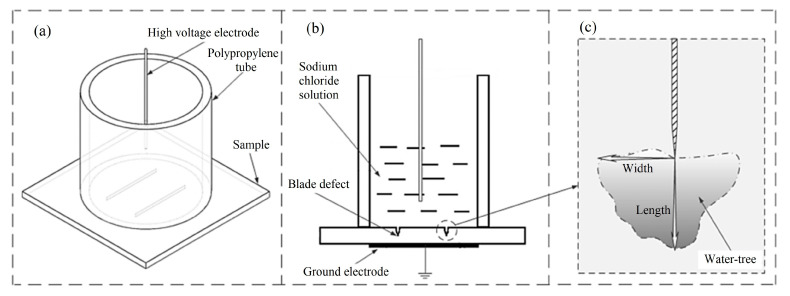
Schematics of accelerated water-tree aging experiments with a water blade electrode: (**a**) overview, (**b**) side sectional view, (**c**) generating water-tree.

Water-tree experiments are fulfilled by accelerating electric aging process with a water blade electrode being continuously applied by AC high voltage of 4 kV at high frequency of 3 kHz for 24–192 h, as schematically shown in Figure 9. The blade electrode in 0.03 mm thickness and with a blade edge in 0.01 mm curvature radius is vertically inserted into the 4 mm thick film sample whilst keeping 2 mm away from the sample’s bottom surface, which causes knife-like defects at blade edge, as shown in Figure 9b. Sodium chloride solution of 1.8 mol/L is used as water medium for water-tree aging experiments. Before water-tree experiment, the entire equipment is degassed in vacuum for 60 min to completely remove residual air in blade defects. After water-tree growth has been finished, the cuboid film sample is sliced (into ~100 μm thickness) through the defects at blade edge along the direction perpendicular to both the blade plane and sample surface, as implemented by the manual rotary microtome (Leica RM2235, Chuangxun Medical Equipment Co., Ltd., Shanghai, China). The sliced samples are then immersed in methylene blue solution persisting at a constant temperature of 90 °C for 4 h, making water-trees legible.

### 3.3. Characterization and Measurement

Infrared absorption spectra are tested for the raw blends and the crosslinked XLPE materials to determine whether or not the grafting process is successful by comparing the characteristic peaks of specific chemical groups before and after crosslinking/grafting reaction process. The film samples in 0.3 mm thickness are tested by Fourier-transform infrared (FTIR) spectrometer (FT/IR-6100, Jiasco Trading Co., Ltd., Shenyang, China) in wavelength range of 500–4000 cm^−1^ with a resolution of 2 cm^−1^.

Differential scanning calorimetry (DSC) is employed to test the heat flow from and out of samples when being gradually heated/cooled at a rate of 5 °C/min in nitrogen atmosphere, as implemented in differential scanning calorimeter (DSC-3, METTLER TOLEDO, Zurich, Switzerland). Polyethylene crystallinity is calculated from enthalpy change in melting process of DSC test, according to *X*_c_ = Δ*H*_m_/Δ*H*_100_, where Δ*H*_100_ = 293.6 J/g and Δ*H*_m_ denote the melting enthalpies of the 100% crystallized material and the tested semi-crystalline samples, respectively.

Water-tree morphology is observed to evaluate water-tree length with an optical microscope (SteREO DiscoveryV20, Carl Zeiss AG, Berlin, Germany). Semi-crystalline morphologies of the grafted and benchmark XLPE materials in 120 μm thick slice samples are observed by polarizing fluorescence microscope (PLM, DM2500P, Leica Co., Berlin, Germany).

Dynamic thermomechanical analysis (DMA) is performed on 15 × 6 × 1 mm^3^ cuboid film samples to evaluate viscoelastic characteristics, as described by energy-storage modulus *E*’, loss modulus *E*” and loss factor tan*θ = E*’/*E*”, in the heating process by a rate of 3 °C/min from −50 to 100 °C under nitrogen atmosphere, as implemented in dynamic thermomechanical analyzer (Q800DMA, TA apparatus Co. Ltd., DE, USA). DMA tests are carried out under tensile stress by specifying target frequency/amplitude and static/dynamic forces of 1 Hz/15 μm and 0.375 N/0.3 N, respectively. Complying with GB/T 1040.2-2006 standard, the stress-strain characteristics of “5A” dumbbell shaped samples with a mark distance of 20 mm in 4 mm width and 2 mm thickness are measured by an elongation speed of 5 mm/min.

### 3.4. Miscibility Computation

Mixing energy, free energy and phase diagram of water (H_2_O) molecules mixing with CAAE or MAH molecules are calculated by Monte Carlo method combined with the modified Flory–Huggins model, using Blends program of Materials Studio 2020 package (Accelrys Inc., Materials Studio version 2020.08, San Diego, CA, USA). According to thermodynamics theory of miscibility and separation in binary phase systems as described by Flory–Huggins model [26,27], the free energy of a binary mixture system is represented as by:

(1) $$\frac{\Delta G}{RT}=\frac{\phi_{b}}{n_{b}}\ln\phi_{b}+\frac{\phi_{s}}{n_{s}}\ln\phi_{s}+\chi \phi_{b}\phi_{s}$$

where Δ*G* denotes mixing free energy (per mole), *ϕ*_i_ and *n*_i_ symbolize volume ratio and polymerization degree of component i respectively, *χ* represents interaction parameter, *T* and *R* indicate absolute temperature and gas constant respectively. The sum of the first two terms are always negative, which is combinatorial entropy in favor of mixed state rather than separating into pure components. The last term of free energy is derived from interaction of mixing different components. Interaction parameter *χ* is defined by *χ*=*E*_mix_/*RT* (*E*_mix_ denotes mixing energy), which indicate free energy difference between mixed and separated phase states, in disfavor of mixing when being positive.

## 4. Conclusions

For the first time, we propose and demonstrate how to improve water-tree aging resistance of XLPE material by grafting polar-group molecules, in which the underlying mechanism is elucidated by the hydrophilia of graft molecules, the crystalline characteristics of heterogeneous nucleation, and the mechanical properties derived from polyethylene molecular-chains in amorphous phase. By means of peroxide-initiated thermochemical grafting method, two species of molecules named by CAAE and MAH, which possess polar-groups, are successfully grafted onto XLPE molecular-chains through free radical addition reactions. Infrared spectroscopy, water-tree aging experiments, crystallization characteristics and mechanical properties are conducted to elucidate the mechanism of improving water resistance by chemically grafting CAAE or MAH molecules. Combined with Monte Carlo molecular simulations, it is verified that water-tree resistance can been significantly promoted by grafting polar-group molecules. It is consistently manifested by DSC spectra, DMA peaks and stress-strain characteristics that the grafted polar groups can enhance Van der Waals’ force between polyethylene molecules and are available as heterogeneous nucleation centers for polyethylene crystallization, which lead to the increased densities of spherulites with the reduced-volume and increased-tenacity amorphous regions between lamellae, accounting for the considerable improvement in water-tree resistance. It is indicated from Monte Carlo molecular simulations that CAAE/H_2_O binary system possesses negative interaction parameters and mixing energies throughout a large temperature range, implying that water molecules will be dispersed without aggregating into water droplets in CAAE grafted XLPE, which alleviates electric-stress impacts on amorphous regions. Inspired by the chemical modifications of XLPE material by grafting polar-group molecules which have been recently verified for improving insulation performances, this paper suggests a feasible strategy of chemical grafts to simultaneously improve water-tree resistances and insulation performances of polyethylene insulation materials in applications of high voltage cable manufactures.

## Figures and Tables

**Table 1:** Melting–crystallization characteristics and the calculated crystallinities.

Material	Melting Peak *T*_m_/°C	Crystallizing Peak *T*_c_/°C	Melting Enthalpy Δ*H*_m_/(J/g)	Crystallinity *X*_c_/%
XLPE	88.76	105.13	98.35	33.5
XLPE-g-1.0wt%CAAE	89.37	106.62	105.11	35.8
XLPE-g-1.0wt%MAH	89.61	106.98	106.29	36.2

**Table 2:** DMA *β* relaxation peak magnitude (loss modulus *E*” and factor tan*θ*), crystallinity *X*_c_ and water-tree dimension (characteristic length *L*_c_).

Material	*E*”/MPa	tan*θ*	*X*_c_/%	*L*_c_/μm
XLPE	81	0.079	33.5	129.8
XLPE-g-1.0wt%CAAE	87	0.085	35.8	80.1
XLPE-g-1.0wt%MAH	90	0.090	36.2	79.6

## Data Availability

The theoretical and experimental methods and results are available from all authors.
